# Evaluation of the effects of a high-efficiency decontamination solution for sodium contamination on sodium burn wounds in rat skin

**DOI:** 10.3389/fbioe.2026.1872380

**Published:** 2026-07-22

**Authors:** Shen Jiang, Teng Zhang, Xiaona Gu

**Affiliations:** Division of Radiology and Environmental Medicine, China Institute for Radiation Protection, Taiyuan, China

**Keywords:** high-efficiency decontamination solution, inflammatory response, pH, sodium burn, sodium contamination, wound repair

## Abstract

**Objective:**

To prepare a compound irrigation formulation for sodium contamination and evaluate its effects on wound pH regulation and early repair outcomes in a rat model of sodium-induced skin injury.

**Methods:**

A compound irrigation formulation for sodium contamination was prepared using glycine and ethylenediaminetetraacetic acid disodium salt as the principal formulation components. Sixty Sprague-Dawley rats were randomly assigned to five groups: no-irrigation group (NI), water irrigation group (WI), boric acid irrigation group (BA), Diphoterine irrigation group (DP), and decontamination solution group (DS), with 12 rats in each group. At 3 min after sodium contamination, wounds were treated with the corresponding interventions and irrigated for 15 min. Wound pH was monitored during irrigation, and wound healing was evaluated on day 14. Histopathological changes were assessed by hematoxylin and eosin (H&E) staining and semi-quantitative injury scoring, and collagen deposition was evaluated by Masson staining. The expression of PCNA, VEGF, α-SMA, and CD31 was detected by immunohistochemistry, and the levels of IL-1β, IL-6, IL-10, and TNF-α were measured by ELISA.

**Results:**

The DS group showed a rapid reduction in wound alkalinity, and wound pH shifted toward a near-neutral range after 15 min of irrigation. Compared with the NI, WI, and BA groups, the DS group had a significantly higher wound healing rate on day 14 (P < 0.05). Histological analysis and semi-quantitative injury scoring showed better tissue preservation, reduced inflammatory cell infiltration, a lower histopathological injury score, and increased collagen deposition in the DS group. PCNA expression did not differ significantly among groups, whereas the expression levels of the angiogenesis- and repair-associated markers VEGF, α-SMA, and CD31 were increased in the DS group compared with the NI and WI groups (P < 0.05). In addition, IL-1β and TNF-α levels were decreased in the DS group compared with the NI and WI groups (P < 0.05), IL-6 was decreased compared with the NI group (P < 0.05), and IL-10 was increased compared with all other groups (P < 0.05).

**Conclusion:**

The compound irrigation formulation rapidly reduced wound alkalinity and was associated with improved early repair outcomes, including increased collagen deposition, enhanced angiogenesis-related marker expression, and a more favorable local inflammatory profile in sodium-induced skin wounds in rats. These findings suggest its potential as an emergency irrigation formulation for sodium contamination, although direct sodium removal and the independent contribution of each component require further investigation.

## 1 Introduction

Sodium-cooled fast reactors (SFRs) are an important type of Generation IV nuclear energy system and have attracted considerable attention because of their high fuel utilization efficiency, favorable thermal performance, and neutron economy (Hill et al., 2011). Liquid metallic sodium is widely used as the primary coolant in SFRs owing to its excellent thermal conductivity and low neutron absorption cross-section (Guidez et al., 2022; Faizal et al., 2023). However, metallic sodium is highly reactive. Leakage or accidental contact during operation, maintenance, or emergency response may lead to sodium contamination of workers and cause a distinctive type of occupational chemical injury (Garti and Coulon, 2021). When metallic sodium comes into contact with water or tissue fluid, it reacts rapidly, releasing a large amount of heat and generating strongly alkaline sodium hydroxide. As a result, sodium-induced skin injury has the combined characteristics of thermal burns and alkali burns. Heat released during the reaction can directly damage local tissues, while the alkaline products can further destroy skin structures through dehydration, lipid saponification, and protein denaturation, with continued penetration into deeper tissues (Walsh et al., 2022; Almarghoub et al., 2020). Therefore, rapid and effective on-site management of sodium contamination is essential not only for improving wound outcomes but also for occupational health and safety in SFR-related and other specialized industrial settings.

At present, first aid for chemical burns mainly relies on prolonged water irrigation, and low-concentration boric acid solution may also be used in some alkali burn scenarios (Chai et al., 2025; Acikel et al., 2001). Water irrigation is readily available and easy to apply, but it mainly acts through mechanical flushing and dilution, and it may require large volumes and prolonged irrigation while being insufficient for removing deeply retained alkaline substances. Boric acid solution can reduce local alkalinity, but acid-base neutralization may be accompanied by heat release, which may further aggravate local tissue injury (Chai et al., 2022; Wang et al., 2023). Diphoterine has been used as an emergency rinsing product for exposure to corrosive or irritant chemicals; however, experimental evidence for its application in metallic sodium contamination, which involves combined thermal and alkali injury, remains limited. Therefore, developing an emergency irrigation formulation with rapid alkalinity reduction, tissue-protective potential, and practical applicability is important for this specific occupational exposure scenario.

Based on the injury characteristics of metallic sodium burns and the need for on-site emergency management, we developed a high-efficiency decontamination solution for sodium contamination. This solution uses glycine (Gly) and ethylenediaminetetraacetic acid disodium salt (EDTA-2Na) as the main decontamination components, with mupirocin and lidocaine included as auxiliary protective components, aiming to establish a compound irrigation system for sodium contamination. The present study focused on evaluating the overall application effects of this compound emergency irrigation formulation rather than the independent pharmacological contribution of each component. Using a rat model of sodium-induced skin injury, we compared the high-efficiency decontamination solution for sodium contamination with water, 3% medical boric acid solution, and Diphoterine, and systematically evaluated its effects on wound pH regulation, wound healing, histopathological changes, collagen deposition, angiogenesis-related markers, and inflammatory factor expression. This study aimed to provide experimental evidence for occupational emergency protection and on-site irrigation strategies after metallic sodium contamination.

## 2 Materials and methods

### 2.1 Animals, reagents, and instruments

Sixty 8-week-old specific pathogen-free female Sprague-Dawley rats, weighing 200 ± 25 g, were purchased from Beijing Vital River Laboratory Animal Technology Co., Ltd (animal production license No. SCXK [Beijing] 2016-0011). All animal procedures were approved by the Experimental Animal Ethics Committee of the China Institute for Radiation Protection (approval No. CIRP-IACUC-CRJ2024024) and were performed in accordance with institutional guidelines for animal welfare.

Rats were housed in a specific pathogen-free animal facility at the China Institute for Radiation Protection under controlled environmental conditions, with a temperature of 22 °C ± 2 °C, relative humidity of 50% ± 10%, and a 12-h light/dark cycle. Animals had free access to standard chow and water and were acclimatized for 7 days before the experiment.

Hematoxylin and eosin (H&E) staining kits, Masson staining kits, immunohistochemical antibodies, and ELISA kits were purchased from Servicebio Technology Co., Ltd (Wuhan, China). The main instruments included a Leica DM1000 LED light microscope (Leica Microsystems GmbH, Germany), a SIGMA3-18K refrigerated high-speed centrifuge (Sigma Laborzentrifugen GmbH, Germany), a LABSERV K3 TOUCH microplate reader (Thermo Fisher Scientific Inc., USA), and a SCIENTZ-12 high-throughput tissue grinder (Ningbo Scientz Biotechnology Co., Ltd., China).

### 2.2 Composition, rationale, preparation, and physicochemical quality control of the high-efficiency decontamination solution for sodium contamination

The high-efficiency decontamination solution for sodium contamination used in this study was designed as a compound irrigation formulation composed of glycine (Gly), ethylenediaminetetraacetic acid disodium salt (EDTA-2Na), mupirocin, and lidocaine. The formulation was developed for rapid on-site irrigation and auxiliary wound protection after metallic sodium exposure, particularly in sodium-cooled fast reactor-related occupational settings. Gly was selected to provide alkalinity buffering, osmotic support for fluid exchange, and wound microenvironment regulation, which may facilitate the removal of residual sodium hydroxide and soluble alkaline products. EDTA-2Na was included as a metal-chelating auxiliary decontamination component to address potentially co-existing multivalent metal ions or metal-containing contaminants, rather than primarily to chelate Na+. Mupirocin and lidocaine were included to provide local antimicrobial and pain-relief support during early wound irrigation, respectively.

Gly and EDTA-2Na were used at final concentrations of 720 mM and 56.6 mM, respectively. Mupirocin and lidocaine were included at final concentrations of 2% and 0.5%, respectively. For the preparation of 1 L solution, 54.05 g Gly, 21.07 g EDTA-2Na, 20 g mupirocin, and 5 g lidocaine were accurately weighed. Approximately 700 mL sterile ultrapure water was added to a beaker and stirred using a magnetic stirrer. Gly and EDTA-2Na were added sequentially and dissolved completely, with ultrasonic treatment applied when necessary to promote dissolution and mixing. Mupirocin and lidocaine were then added, and stirring was continued until a homogeneous formulation was obtained. The mixture was transferred to a 1000 mL volumetric flask, diluted to volume with sterile ultrapure water, mixed thoroughly, and dispensed into sterile sealed containers. The final product was stored protected from light until use.

The finished solution was examined for appearance, color, clarity, and visible foreign matter. Physicochemical and quality-control properties, including pH, stability, heavy metal content, and total bacterial colony count, were also evaluated. For stability testing, samples were stored at 40 °C and −5 °C for 24 h, respectively, and then returned to room temperature for observation of stratification, discoloration, odor, crystallization, or phase separation.

### 2.3 Establishment and characterization of the rat model of sodium-induced skin injury

Zoletil 50, a tiletamine-zolazepam injectable anesthetic, was used for anesthesia. Rats were anesthetized by subcutaneous injection of Zoletil 50 (tiletamine-zolazepam) at a dose of 0.06 mL/100 g body weight, corresponding to 30 mg/kg based on a reconstituted total concentration of 50 mg/mL. The depth of anesthesia was assessed by the absence of spontaneous movement and pedal withdrawal reflex before sodium exposure. Dorsal hair was removed, and the skin was disinfected with 75% medical alcohol. The skin surface was allowed to dry completely before sodium exposure to avoid premature reaction between metallic sodium and residual liquid. Metallic sodium stored in kerosene was cut into blocks with an approximate contact area of 1 cm^2^ immediately before use, and residual kerosene was carefully removed with dry gauze. To improve model consistency, the contact area of the sodium block and the exposure duration were kept consistent across animals. The sodium block was applied to the dorsal skin of each rat for 3 min without additional external pressure. After exposure, the sodium block was removed, and residual metallic sodium on the wound surface was carefully cleared using dry forceps to establish a rat model of sodium-induced skin injury.

The 60 rats were randomly assigned to five groups, with 12 rats in each group: no-irrigation group (NI), water irrigation group (WI), boric acid irrigation group (BA), Diphoterine irrigation group (DP), and decontamination solution group (DS). After model establishment, rats in the NI group received no irrigation; rats in the WI group were irrigated with water; rats in the BA group were irrigated with 3% medical boric acid solution; rats in the DP group were irrigated with Diphoterine; and rats in the DS group were irrigated with the high-efficiency decontamination solution for sodium contamination. To standardize the intervention procedure, irrigation was started immediately after removal of residual metallic sodium and was performed continuously for 15 min using a peristaltic pump at a flow rate of 50 mL/min. The irrigation distance and direction were kept consistent among animals as far as possible.

On day 14 after sodium contamination, rats were deeply anesthetized with Zoletil 50 as described above and euthanized by cervical dislocation under deep anesthesia. Death was confirmed by the absence of respiration, heartbeat, and pain reflexes. Skin wound tissues were then collected. Some tissues were fixed in 4% paraformaldehyde for histological and immunohistochemical analyses, whereas the remaining tissues were stored at −80 °C for subsequent ELISA.

### 2.4 Dynamic monitoring of wound pH, gross wound observation, and wound healing rate measurement

After establishment of the rat model of sodium-induced skin injury, wound pH was measured using a portable skin pH meter before irrigation and at 15 s, 30 s, 1 min, 3 min, 5 min, 10 min, and 15 min after the start of irrigation. After irrigation, wound photographs were taken to record gross morphological changes.

Wound healing was observed daily. Photographs were taken on days 1, 3, 7, 10, and 14 after sodium contamination using a fixed shooting angle and distance, with a ruler placed as a scale reference. Wound areas were measured using Image-Pro Plus software from coded wound photographs by an investigator blinded to group allocation. The wound healing rate on day 14 was calculated as follows: Wound healing rate = (initial wound area − unhealed wound area)/initial wound area × 100%.

### 2.5 H&E staining and semi-quantitative histopathological injury scoring

Skin wound tissues fixed in 4% paraformaldehyde were embedded in paraffin and sectioned into 4-μm-thick slices. The sections were baked at 65 °C for 1 h, deparaffinized in xylene, and rehydrated through graded ethanol. H&E staining was then performed. After dehydration, clearing, and mounting, the sections were observed under a light microscope to evaluate skin tissue integrity, tissue necrosis, inflammatory cell infiltration, and dermal structural changes.

Histopathological injury was further assessed using a semi-quantitative scoring system based on H&E-stained sections (Guo et al., 2020; Gupta and Kumar, 2015). Four parameters were evaluated: epidermal disruption, tissue necrosis, inflammatory cell infiltration, and dermal structural disorganization. Each parameter was scored from 0 to 3, with higher scores indicating more severe injury. The total histopathological injury score ranged from 0 to 12. For each rat, three non-overlapping fields were evaluated, and the mean value was used for statistical analysis.

Before evaluation, all histological images were coded to mask group allocation and treatment information. Histopathological evaluation and scoring were independently performed by two external pathologists who were not involved in the experimental design or animal treatment and were blinded to group allocation and treatment information. The final score for each rat was calculated as the mean score of the two pathologists.

### 2.6 Masson staining for collagen deposition

Paraffin sections of skin wound tissues were deparaffinized and rehydrated, followed by Masson staining according to the manufacturer’s instructions. Collagen fibers were stained blue and observed under a light microscope. Image-Pro Plus software was used to quantify the blue-stained collagen area, and the percentage of collagen deposition area was calculated.

### 2.7 Immunohistochemical detection of PCNA, VEGF, α-SMA, and CD31

Paraffin sections of wound tissues from each group were deparaffinized and rehydrated. Antigen retrieval was performed using EDTA antigen retrieval solution (pH 8.0). Endogenous peroxidase activity was blocked by incubation with 3% hydrogen peroxide for 25 min at room temperature in the dark. After blocking nonspecific binding sites, sections were incubated overnight at 4 °C with primary antibodies against proliferating cell nuclear antigen (PCNA), vascular endothelial growth factor (VEGF), α-smooth muscle actin (α-SMA), and platelet endothelial cell adhesion molecule-1 (PECAM-1/CD31). The next day, sections were incubated with the corresponding secondary antibodies at room temperature for 50 min. DAB was used for color development, and hematoxylin was used for nuclear counterstaining. After dehydration, clearing, and mounting, the sections were observed and photographed under a light microscope.

In immunohistochemical staining, positive DAB staining appeared brown-yellow, while nuclei counterstained with hematoxylin appeared blue. Image-Pro Plus software was used to measure the average optical density of PCNA-, VEGF-, and α-SMA-positive staining areas to reflect the expression levels of the corresponding proteins. CD31 staining was used to evaluate neovascularization, and the average microvessel density (MVD) was calculated from three non-overlapping fields for each sample. In addition, immunohistochemical sections were qualitatively reviewed by the same two external pathologists using coded images, and the pathologists were blinded to group allocation and treatment information.

### 2.8 ELISA detection of inflammatory factors in wound tissues

Skin wound tissues from each group were accurately weighed and mixed with 0.9% saline at a tissue weight (mg) to saline volume (μL) ratio of 1:9. The tissues were mechanically homogenized in an ice-water bath to prepare 10% tissue homogenates. The homogenates were centrifuged at 2,500–3,000 rpm for 10 min, and the supernatants were collected for analysis.

The levels of tumor necrosis factor-α (TNF-α), interleukin-1β (IL-1β), interleukin-6 (IL-6), and interleukin-10 (IL-10) in wound tissues were measured using ELISA kits according to the manufacturer’s instructions. Within 10 min after termination of the reaction, optical density values were measured at 450 nm using a microplate reader. Standard curves were generated based on the concentrations and optical density values of the standards, and the levels of inflammatory factors in the samples were calculated accordingly.

### 2.9 Statistical analysis

Statistical analysis was performed using R software version 4.0.0, and graphs were generated using GraphPad Prism 10.1. The normality of data distribution was assessed using the Shapiro-Wilk test, and homogeneity of variance was evaluated using Levene’s test. Quantitative data that met the assumptions of normal distribution and homogeneity of variance are expressed as mean ± SEM. Comparisons among multiple groups were performed using one-way analysis of variance (one-way ANOVA), followed by Tukey’s multiple comparisons test. For data that did not meet these assumptions, the Kruskal–Wallis test followed by Dunn’s multiple comparisons test was used. Histopathological injury scores were considered semi-quantitative ordinal data and were analyzed using the Kruskal–Wallis test followed by Dunn’s multiple comparisons test. These scores are presented as box-and-whisker plots with individual data points. The sample size of 12 rats per group was selected based on previous wound-healing animal studies, preliminary experimental observations, and animal welfare considerations. A P value <0.05 was considered statistically significant.

## 3 Results

### 3.1 Properties and quality evaluation of the high-efficiency decontamination solution for sodium contamination

At room temperature, the finished high-efficiency decontamination solution for sodium contamination appeared as a colorless, transparent, and clear liquid, with no obvious odor or visible foreign matter. Stability testing showed that the solution exhibited no stratification, discoloration, or odor after storage at 40 °C for 24 h. After storage at −5 °C for 24 h and subsequent return to room temperature, no crystallization, phase separation, or precipitation was observed. Quality testing showed that the levels of lead, mercury, arsenic, cadmium, and chromium were below the detection limits, the total bacterial colony count was <20 CFU/mL, and the pH of the solution was 5.98. These results indicated favorable appearance stability and basic quality control characteristics of the solution (Table 1).

**TABLE 1:** Quality control results of the high-efficiency decontamination solution for sodium contamination.

No.	Test item	Test result	Unit	Test method
1	Lead	<0.09	mg/kg	Safety and technical standards for cosmetics
2	Mercury	<0.0033	mg/kg	Safety and technical standards for cosmetics
3	Arsenic	<0.0033	mg/kg	Safety and technical standards for cosmetics
4	Cadmium	<0.0033	mg/kg	Safety and technical standards for cosmetics
5	Chromium	<0.05	mg/kg	Safety and technical standards for cosmetics
6	Total bacterial colony count	<20	CFU/mL	Technical standard for disinfection
7	pH	5.98	-	Technical standard for disinfection

### 3.2 Effects of the high-efficiency decontamination solution for sodium contamination on gross wound appearance, wound healing rate, and wound pH

**FIGURE 1:**
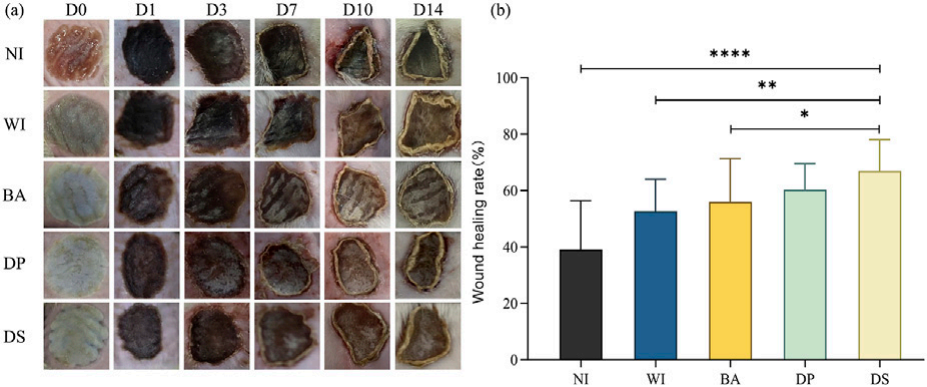
Gross appearance of skin wounds **(a)** and wound healing rate **(b)** in each group of rats. **(a)** Gross appearance of skin wounds on days 1, 3, 7, 10, and 14 after sodium contamination. **(b)** Wound healing rate on day 14. Data are expressed as mean ± SEM; n = 12 rats/group. Data were analyzed by one-way ANOVA followed by Tukey’s multiple comparisons test. ∗P < 0.05 versus the NI, WI, and BA groups. NI, no-irrigation group; WI, water irrigation group; BA, boric acid irrigation group; DP, Diphoterine irrigation group; DS, decontamination solution group treated with the high-efficiency decontamination solution for sodium contamination.

Gross observation showed that after exposure to metallic sodium, wounds in the NI group developed a mucus-like gelatinous layer on the surface, accompanied by marked surrounding redness and swelling. After irrigation, the WI, BA, DP, and DS groups showed varying degrees of reduction in local redness and swelling, and the wound color in the DP and DS groups was lighter than that in the WI and BA groups. On day 10 after sodium contamination, partial scab detachment with slight bleeding was observed in the NI and WI groups. On day 14, the scabs in the DP and DS groups were thinner and the wound margins were clearer than those in the other groups (Figure 1a). Dynamic observation of wound area showed a decreasing trend during the first 7 days after sodium contamination in all groups. Quantitative analysis on day 14 showed that the wound healing rate was significantly higher in the DS group than in the NI, WI, and BA groups (P < 0.05; Figure 1b).

**FIGURE 2:**
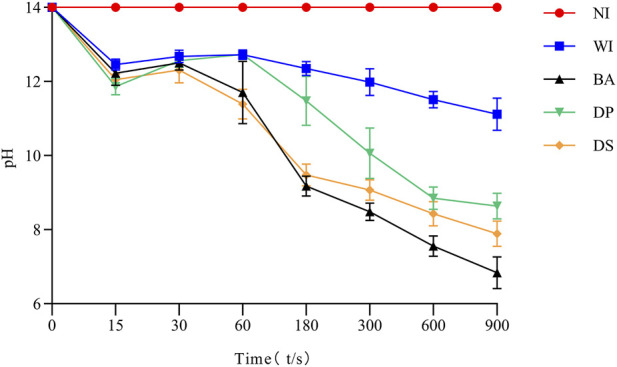
Changes in skin wound pH over irrigation time in each group of rats. Wound pH was measured before irrigation and at 15 s, 30 s, 1 min, 3 min, 5 min, 10 min, and 15 min after the start of irrigation following sodium contamination. Data are expressed as mean ± SEM.

Dynamic monitoring of wound pH showed that the pH in the NI group remained strongly alkaline during the 15 min observation period because no irrigation was performed. In contrast, wound pH decreased to varying degrees in the WI, BA, DP, and DS groups within 1–5 min after the start of irrigation. At 15 min, the wound pH values in the NI, WI, BA, DP, and DS groups were 14.0, 11.1, 6.8, 8.6, and 7.8, respectively. Compared with the WI group, the DS group showed a more pronounced reduction in wound pH. In direct comparison with the DP group, the final wound pH in the DS group was closer to a near-neutral range. Compared with the BA group, DS treatment avoided a further shift of wound pH toward a more acidic range. These results indicate that DS treatment had favorable wound pH-regulating capacity, although direct sodium removal was not quantified in this study (Figure 2).

### 3.3 Effects of the high-efficiency decontamination solution for sodium contamination on histopathological changes and semi-quantitative injury scoring in skin wounds

**FIGURE 3:**
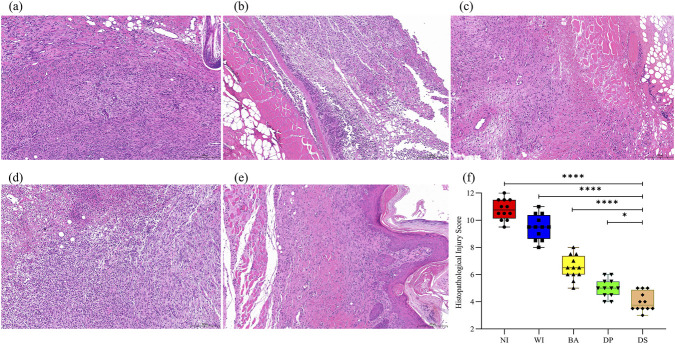
Histopathological changes and semi-quantitative injury scoring of skin wounds in each group of rats. H&E staining of skin wound tissues in each group on day 14 after sodium contamination. **(a)** NI; **(b)** WI; **(c)** BA; **(d)** DP; **(e)** DS. Scale bar = 200 μm. **(f)** Semi-quantitative histopathological injury score based on H&E-stained sections. The score was calculated according to epidermal disruption, tissue necrosis, inflammatory cell infiltration, and dermal structural disorganization. Each point represents one rat. Data are presented as box-and-whisker plots with individual data points; n = 12 rats/group. Data were analyzed using the Kruskal–Wallis test followed by Dunn’s multiple comparisons test. ^∗^P < 0.05, ^∗∗∗∗^P < 0.0001.

H&E staining showed necrosis of the epidermis and superficial dermis in the NI and WI groups, accompanied by marked fibrous connective tissue proliferation and extensive inflammatory cell infiltration, mainly consisting of lymphocytes and granulocytes. Abundant neovascularization, focal epidermal thickening at the wound margins, and occasional blister formation were also observed. Compared with the NI and WI groups, the BA group showed milder tissue injury, reduced inflammatory cell infiltration, and less structural disruption. The DP group still showed fibrous connective tissue proliferation, neovascularization, and inflammatory cell infiltration to some extent, but the overall degree of tissue damage was reduced compared with the NI and WI groups. In the DS group, skin tissue structure was relatively well preserved, keratinization was increased, epidermal continuity and integrity were improved, and inflammatory cell infiltration was reduced, indicating milder overall histopathological damage (Figures 3a–e).

Semi-quantitative histopathological injury scoring further supported the descriptive histological findings. The DS group showed the lowest histopathological injury score among the five groups. Compared with the NI, WI, and BA groups, the DS group showed a significantly lower histopathological injury score (P < 0.0001). The histopathological injury score was also lower in the DS group than in the DP group (P < 0.05). These results indicated that DS treatment was associated with reduced histopathological injury in sodium-induced skin wounds (Figure 3F).

### 3.4 Effects of the high-efficiency decontamination solution for sodium contamination on collagen deposition in skin wounds

**FIGURE 4:**
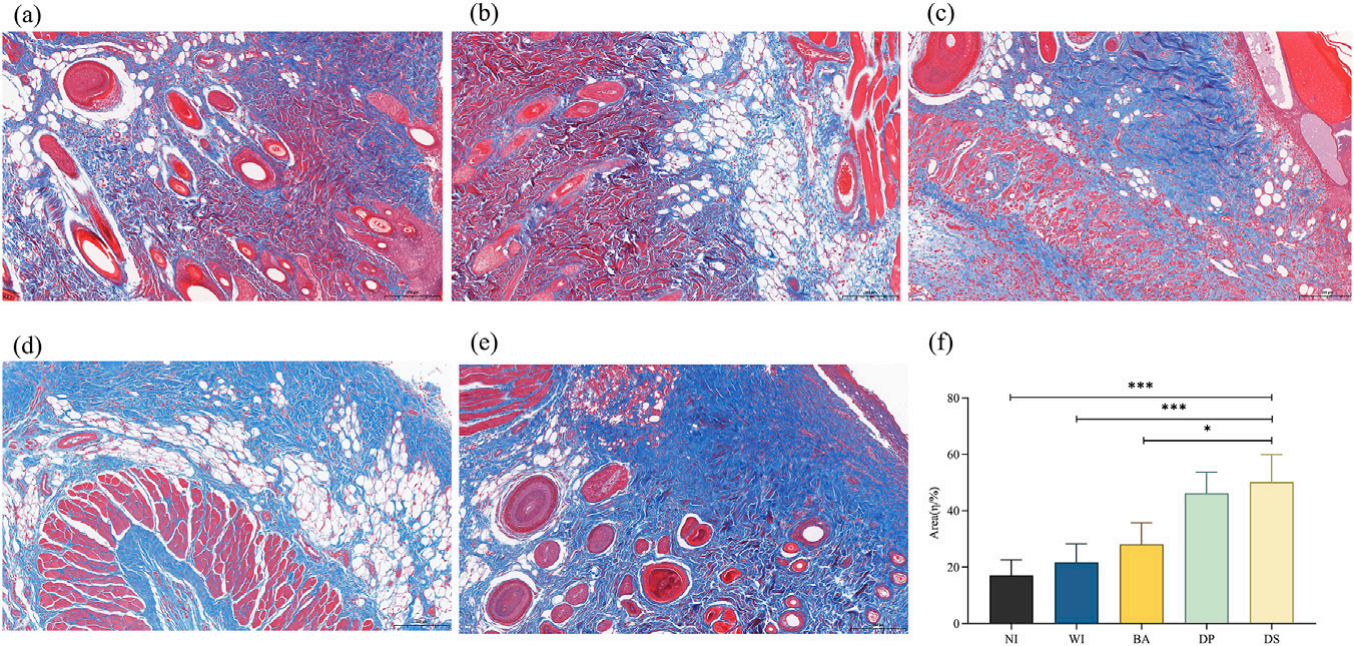
Collagen deposition in skin wound tissues in each group of rats. Masson staining of skin wound tissues on day 14 after sodium contamination. Blue-stained areas indicate collagen fibers. **(a)** NI; **(b)** WI; **(c)** BA; **(d)** DP; **(e)** DS. Scale bar = 200 μm. **(f)** Quantitative analysis of collagen deposition area. Data are expressed as mean ± SEM; n = 12 rats/group, three non-overlapping fields/rat were analyzed, and the mean value per rat was used for statistics. Data were analyzed by one-way ANOVA followed by Tukey’s multiple comparisons test. ^∗^P < 0.05, ^∗∗∗^P < 0.001.

Masson staining showed relatively loose collagen fiber arrangement and structural disorganization in some areas of the NI and WI groups, whereas collagen deposition was improved in the BA group. In the DP and DS groups, blue-stained collagen fibers were increased, fiber bundles were clearer, and collagen fibers were more closely connected and more orderly arranged (Figures 4a–e). Quantitative analysis showed that the collagen deposition area was significantly increased in the DP and DS groups compared with the NI, WI, and BA groups (P < 0.05 and P < 0.001; Figure 4f). These findings indicated that DS treatment was associated with increased collagen deposition and improved dermal tissue reconstruction.

### 3.5 Effects of the high-efficiency decontamination solution for sodium contamination on PCNA, VEGF, α-SMA, and CD31 expression in skin wounds

**FIGURE 5:**
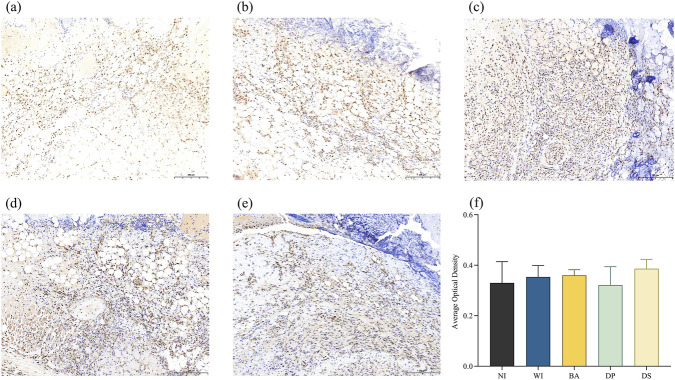
Expression of PCNA in skin wound tissues in each group of rats. Immunohistochemical staining of PCNA in skin wound tissues on day 14 after sodium contamination. Brown-yellow staining indicates positive PCNA expression. **(a)** NI; **(b)** WI; **(c)** BA; **(d)** DP; **(e)** DS. Scale bar = 200 μm. **(f)** Quantitative analysis of PCNA expression. Data are expressed as mean ± SEM; n = 12 rats/group, three non-overlapping fields/rat were analyzed, and the mean value per rat was used for statistics. Data were analyzed by one-way ANOVA followed by Tukey’s multiple comparisons test. Ns, not significant.

**FIGURE 6:**
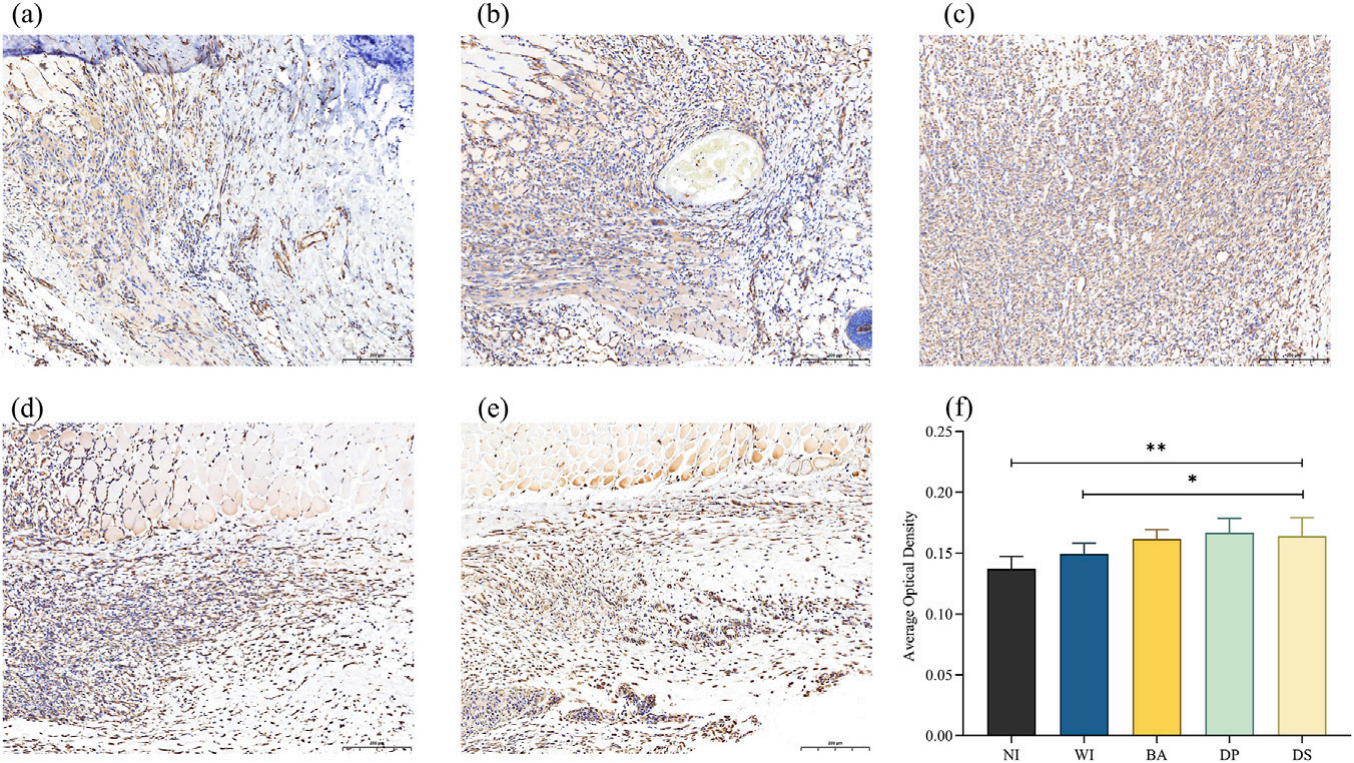
Expression of VEGF in skin wound tissues in each group of rats. Immunohistochemical staining of VEGF in skin wound tissues on day 14 after sodium contamination. Brown-yellow staining indicates positive VEGF expression. **(a)** NI; **(b)** WI; **(c)** BA; **(d)** DP; **(e)** DS. Scale bar = 200 μm. **(f)** Quantitative analysis of VEGF expression. Data are expressed as mean ± SEM; n = 12 rats/group, three non-overlapping fields/rat were analyzed, and the mean value per rat was used for statistics. Data were analyzed by one-way ANOVA followed by Tukey’s multiple comparisons test. ^∗^P < 0.05, ^∗∗^P < 0.01.

Immunohistochemical staining showed varying degrees of PCNA-positive expression in skin wound tissues of all groups, but no significant difference was observed among the groups (P > 0.05; Figures 5a–f). VEGF immunohistochemical staining showed stronger VEGF-positive expression in the DS group than in the NI and WI groups. Quantitative analysis confirmed that VEGF expression was significantly higher in the DS group than in the NI and WI groups (P < 0.05 and P < 0.01; Figures 6a–f).

**FIGURE 7:**
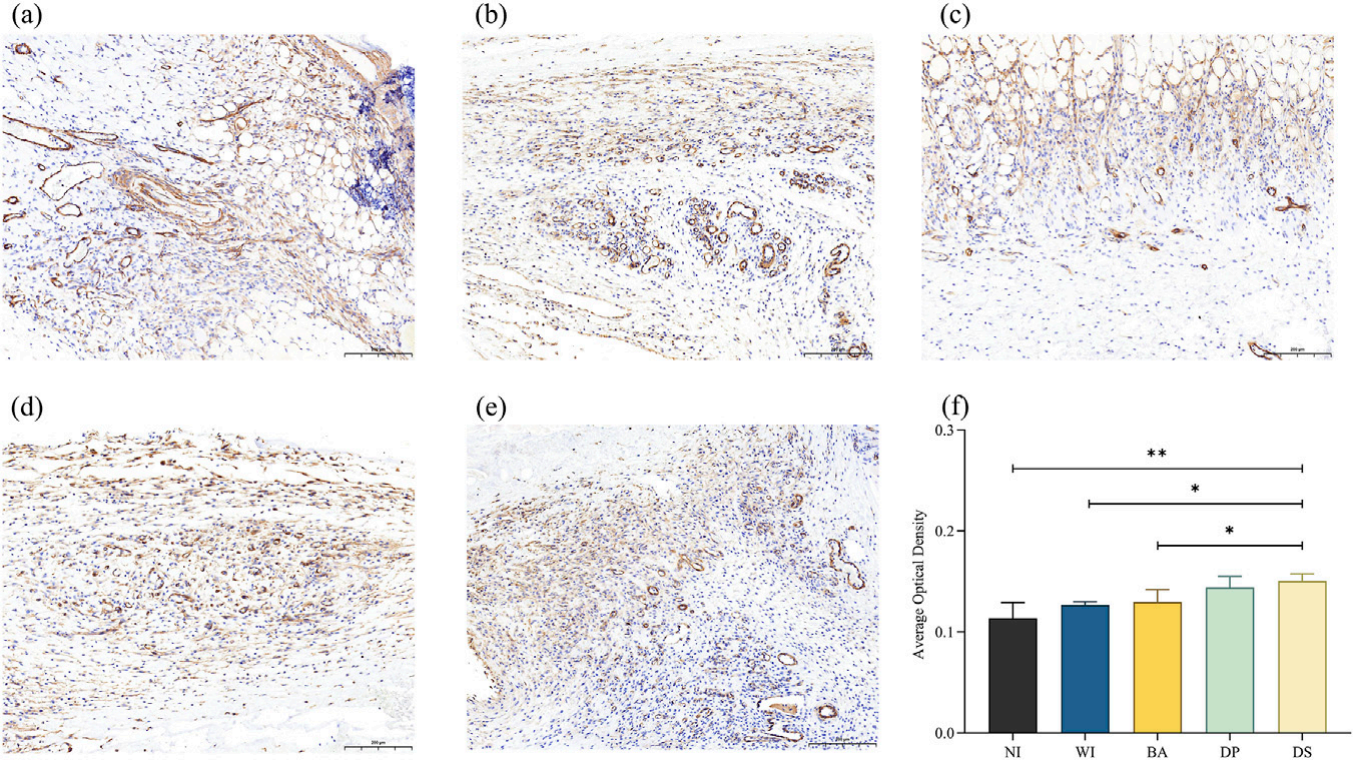
Expression of α-SMA in skin wound tissues in each group of rats. Immunohistochemical staining of α-SMA in skin wound tissues on day 14 after sodium contamination. Brown-yellow staining indicates positive α-SMA expression. **(a)** NI; **(b)** WI; **(c)** BA; **(d)** DP; **(e)** DS. Scale bar = 200 μm. **(f)** Quantitative analysis of α-SMA expression. Data are expressed as mean ± SEM; n = 12 rats/group, three non-overlapping fields/rat were analyzed, and the mean value per rat was used for statistics. Data were analyzed by one-way ANOVA followed by Tukey’s multiple comparisons test. ^∗^P < 0.05, ^∗∗^P < 0.01.

**FIGURE 8:**
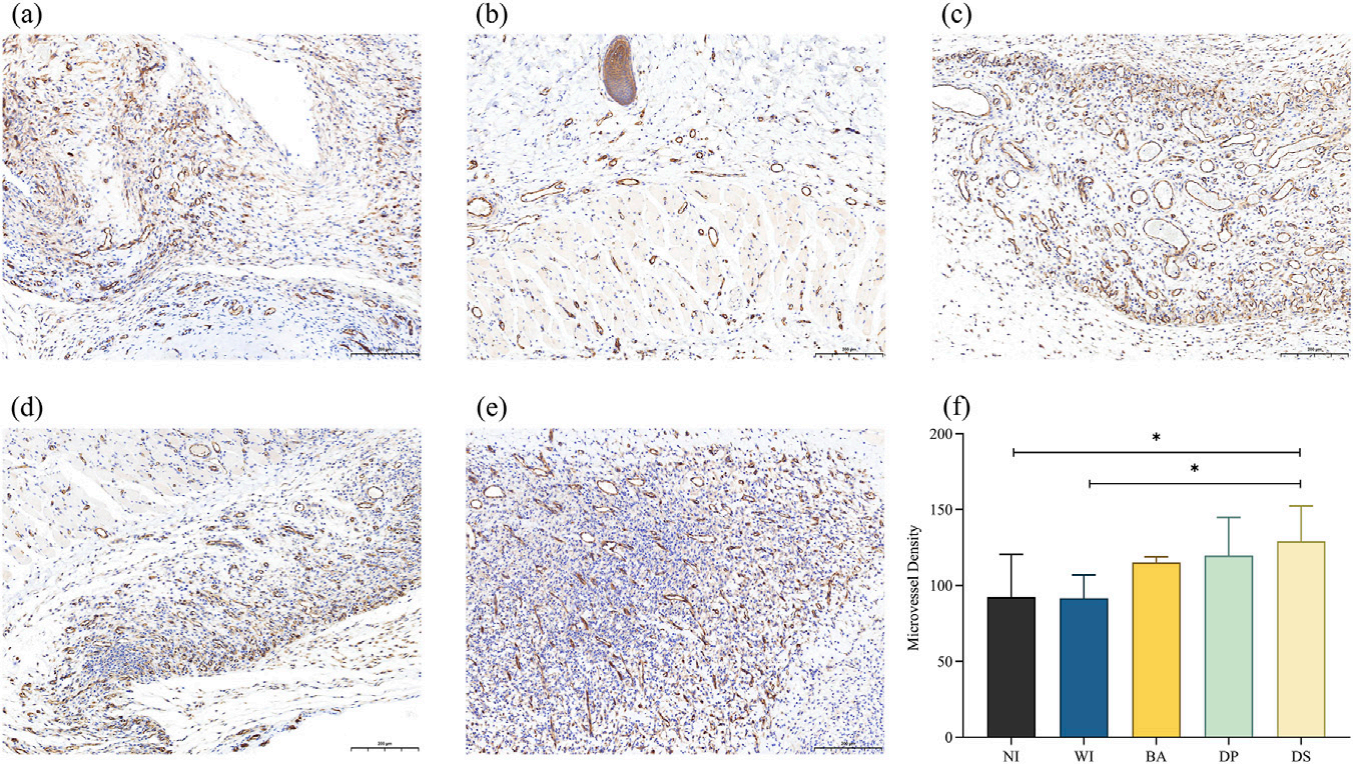
Expression of CD31 in skin wound tissues in each group of rats. Immunohistochemical staining of CD31 in skin wound tissues on day 14 after sodium contamination. Brown-yellow staining indicates positive CD31 expression. **(a)** NI; **(b)** WI; **(c)** BA; **(d)** DP; **(e)** DS. Scale bar = 200 μm. **(f)** Quantitative analysis of CD31-positive neovessels. Data are expressed as mean ± SEM; n = 12 rats/group, three non-overlapping fields/rat were analyzed, and the mean value per rat was used for statistics. Data were analyzed by one-way ANOVA followed by Tukey’s multiple comparisons test. ^∗^P < 0.05.

α-SMA immunohistochemical staining showed enhanced α-SMA-positive expression in the DS group compared with the NI, WI, and BA groups. Quantitative analysis showed that α-SMA expression was significantly increased in the DS group compared with the NI, WI, and BA groups (P < 0.05 and P < 0.01; Figures 7a-f). CD31 immunohistochemical staining showed an increased number of CD31-positive neovessels in the DS group. Quantitative analysis demonstrated that the number of CD31-positive neovessels was significantly higher in the DS group than in the NI and WI groups (P < 0.05; Figures 8a-f). These results suggested that DS treatment enhanced angiogenesis-related marker expression and may be associated with improved skin wound repair.

### 3.6 Effects of the high-efficiency decontamination solution for sodium contamination on inflammatory factor levels in skin wounds

**FIGURE 9:**
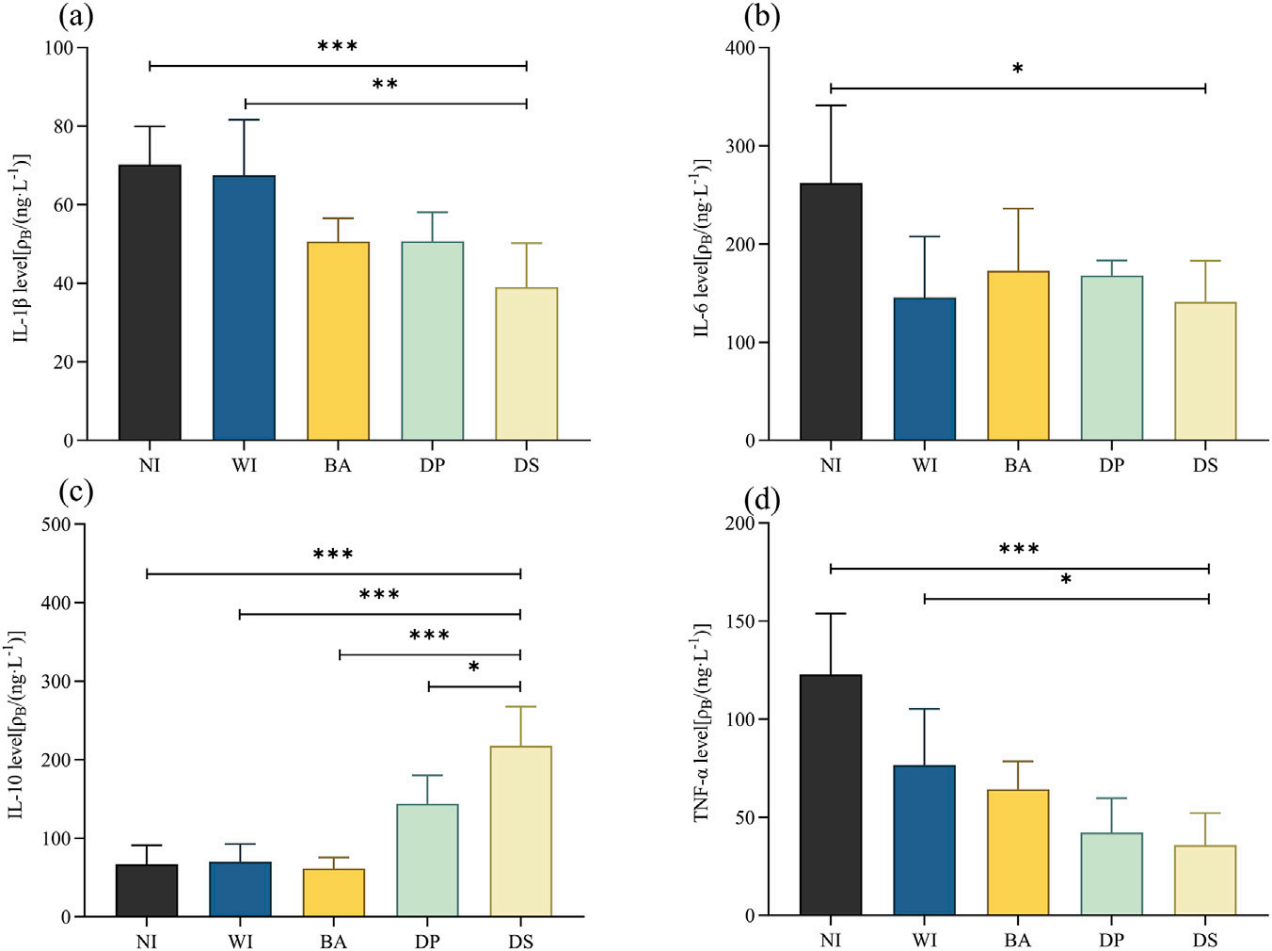
Levels of inflammatory factors in skin wound tissues in each group of rats. The levels of inflammatory factors in skin wound tissues on day 14 after sodium contamination were measured by ELISA. **(a)** IL-1β; **(b)** IL-6; **(c)** IL-10; **(d)** TNF-α. Data are expressed as mean ± SEM; n = 12 rats/group. Data were analyzed by one-way ANOVA followed by Tukey’s multiple comparisons test. ^∗^P < 0.05, ^∗∗^P < 0.01, ^∗∗∗^P < 0.001.

ELISA results showed that the levels of the pro-inflammatory factors IL-1β and TNF-α were significantly decreased in the DS group compared with the NI and WI groups (P < 0.05, P < 0.01, and P < 0.001; Figures 9a,d). Compared with the NI group, the DS group also showed a significant reduction in IL-6 level (P < 0.05; Figure 9b). In contrast, the level of the anti-inflammatory factor IL-10 was significantly higher in the DS group than in the NI, WI, BA, and DP groups (P < 0.05 and P < 0.001; Figure 9c). These findings indicated that DS treatment was associated with a more favorable local inflammatory profile, as reflected by reduced pro-inflammatory factor levels and increased anti-inflammatory factor expression.

## 4 Discussion

Metallic sodium burns have the dual injury characteristics of thermal burns and alkali burns. After contact with the skin, metallic sodium reacts violently with water in tissue cells and releases a large amount of heat, resulting in thermal injury. Meanwhile, the generated strongly alkaline sodium hydroxide can destroy tissue structures through dehydration and lipid saponification and can continuously penetrate into deeper tissues. The severity of burns is mainly evaluated according to burn area and depth. Burn area is usually expressed as the percentage of the total body surface area involved, while burn depth is commonly classified using the three-degree and four-level clinical grading system (Moore et al., 2026; Devgan et al., 2006).

For alkali burns, early and sufficient irrigation is a key first-aid measure. Its core purpose is to rapidly remove alkaline substances from the wound surface and deep tissues and restore the local microenvironmental pH as soon as possible, thereby effectively preventing further invasion of alkaline substances into deeper tissues, reducing injury depth, and improving prognosis (Tan and Wong, 2015). At present, neutralizing solutions are generally not recommended in clinical practice. After wound cleansing, exposure therapy is often adopted, while debridement and skin grafting should be performed as early as possible for deep burns (Jung et al., 2020). During wound repair, inflammatory response, collagen deposition, and epithelial regeneration are key processes and are closely associated with scar formation (Rea et al., 2009). After burns, the skin barrier is damaged and susceptible to secondary infection, while excessive inflammation caused by infection can significantly delay wound healing and inhibit neovascularization (Church et al., 2006). Therefore, during emergency treatment, achieving rapid restoration of wound pH while also considering inflammatory regulation and tissue regeneration has become an important research direction for promoting wound healing and improving prognosis.

As a commonly used chelating agent, EDTA is widely applied in cosmetics and medical formulations and has good biocompatibility, with low skin irritation at conventional use concentrations. Although EDTA cannot directly chelate monovalent sodium ions, it can effectively bind transition metal ions *in vivo*, such as Fe^2+^ and Cu^2+^, thereby inhibiting free radical generation, reducing lipid peroxidation, protecting the integrity of cell membrane structures, and alleviating the progression of tissue necrosis after burns to a certain extent. In addition, disodium EDTA has weak acidity and may participate in regulating solution pH. Together with glycine, it may contribute to local alkalinity reduction and wound pH regulation, but its specific contribution to sodium removal was not directly determined in the present study (Lanigan and Yamarik, 2002; El Ayadi et al., 2021; Wills, 1966).

Glycine is a naturally occurring amino acid with favorable biosafety and tissue compatibility. Its weakly acidic characteristics may help buffer a strongly alkaline microenvironment, and its involvement in collagen synthesis may support repair-associated extracellular matrix formation (Li and Wu, 2018). In this study, the compound irrigation formulation was prepared using EDTA-2Na and glycine as principal formulation components, with lidocaine and mupirocin included as auxiliary protective components. Therefore, the observed biological effects should be interpreted as the overall effect of the compound formulation rather than the isolated action of any single ingredient. In the rat model of sodium-induced skin injury, DS treatment reduced wound pH within 15 min and increased the wound healing rate on day 14. These findings suggest that improved wound pH regulation and a more favorable repair microenvironment may contribute to early tissue repair; however, direct removal of sodium or residual alkaline substances was not quantified in this study.

During wound repair, the formation of granulation tissue depends on the combined effects of type III collagen secreted by fibroblasts and newly formed capillaries, which gradually fill the tissue defect. Subsequently, type III collagen is gradually replaced by type I collagen, thereby enhancing tissue mechanical strength and providing structural support for epithelial cell migration and proliferation (Prost-Squarcioni et al., 2008; Huang et al., 2022). In this study, VEGF and CD31 expression levels in wound tissues were increased after DS treatment, suggesting enhanced angiogenesis-related responses. However, functional angiogenesis was not directly assessed. α-SMA is a marker of myofibroblasts, and its increased expression is generally associated with wound contraction and tissue remodeling (Hinz, 2016). The increased collagen deposition and elevated α-SMA expression observed in the DS group suggest that DS treatment may be associated with improved fibrous tissue reconstruction and repair-related remodeling.

Inflammatory response plays an important regulatory role in the repair process of sodium-contaminated wounds. Moderate inflammation helps remove necrotic tissue and pathogens, whereas excessive inflammation delays wound healing. Sodium burns disrupt the skin barrier and create open wounds, which are prone to infection and can induce high expression of pro-inflammatory factors such as IL-1β, TNF-α, and IL-6, thereby inhibiting tissue repair (Qian et al., 2016). The results of this study showed that after DS treatment, the expression levels of IL-1β, TNF-α, and IL-6 in wound tissues were decreased, while the expression level of the anti-inflammatory factor IL-10 was increased. These findings suggest that DS treatment was associated with a more favorable inflammatory microenvironment, which may have contributed to the observed improvement in early wound repair.

Our study had several limitations. First, the compound irrigation formulation contained multiple biologically active components, including glycine, EDTA-2Na, mupirocin, and lidocaine; therefore, the independent contribution of each component could not be determined. Second, wound pH was used as an indirect indicator of residual alkalinity and wound microenvironment regulation, whereas tissue sodium burden, residual alkali content, and actual chemical removal efficiency were not directly quantified. Third, although the standardized rat model allowed controlled comparison among different irrigation strategies, it may not fully reproduce the complexity of real-world occupational sodium exposure, such as delayed irrigation, larger contaminated areas, persistent contamination, protective clothing interaction, and mixed thermal-chemical injury. In addition, the observation period was limited to 14 days, and long-term scar maturation, fibrosis, tissue remodeling, functional recovery, and repeated-exposure safety were not evaluated. Future studies incorporating direct sodium removal assessment, longer follow-up periods, expanded safety testing, and more realistic exposure models are needed to further clarify the mechanistic and translational value of this compound irrigation formulation.

## 5 Conclusion

In this study, a compound irrigation formulation for sodium contamination was prepared and evaluated in a rat model of sodium-induced skin injury. DS treatment rapidly reduced wound alkalinity during the early irrigation period and shifted wound pH toward a near-neutral range. DS treatment was also associated with an increased wound healing rate on day 14, milder histopathological injury, increased collagen deposition, enhanced angiogenesis-related marker expression, and a more favorable local inflammatory profile in wound tissues.

These findings suggest that the compound irrigation formulation may improve wound pH regulation and early repair outcomes in sodium-induced skin wounds in rats. Further studies are warranted to optimize the formulation and evaluate its mechanistic basis, long-term safety, and translational applicability in occupational emergency management of metallic sodium contamination.

## Data Availability

The original contributions presented in the study are included in the article/supplementary material, further inquiries can be directed to the corresponding author.
